# Per-Layer UV Exposure-Dependent Cytotoxicity of 3D-Printed Aligner Resins and Estrogen-like Activity Screening

**DOI:** 10.3390/polym18202469

**Published:** 2026-10-10

**Authors:** Luka Šimunović, Antun Jakob Marić, Senka Meštrović

**Affiliations:** 1 Department of Orthodontics, School of Dental Medicine, University of Zagreb, 10000 Zagreb, Croatia; mestrovic@sfzg.hr; 2 Faculty of Mechanical Engineering and Naval Architecture, University of Zagreb, 10000 Zagreb, Croatia; antun@3dtech.hr

**Keywords:** 3D printing, clear aligners, cytotoxicity, per-layer UV exposure, photopolymerization, estrogen-like activity, orthodontic polymers

## Abstract

Per-layer UV exposure during 3D printing may influence polymerization and the biological response of photocurable aligner resins. This study evaluated exposure-dependent cytotoxicity of Tera Harz TC-85 and NextDent Ortho Flex and performed an exploratory screening of selected resin-specific printing conditions for ER-selective estrogen-like responses. TC-85 was printed at 2.0, 2.4, 3.0, and 4.0 s per layer, and Ortho Flex at 5.0, 6.0, 7.0, and 8.0 s per layer. The resin-specific per-layer UV exposure ranges were selected based on our previous physicochemical characterization of the same materials and printing system. Extracts were prepared according to ISO 10993-12. Cytotoxicity was assessed in human gingival fibroblasts using the MTT assay, while selected conditions (TC-85, 2.4 s; Ortho Flex, 7.0 s) were evaluated in estrogen receptor (ER)-positive MCF-7 and ER-negative MDA-MB-231 cells as an exploratory screening assay rather than a direct test of ER activation. For both resins, per-layer exposure condition, extract concentration, and their interaction significantly affected the normalized MTT response. At 50% extract concentration, normalized MTT responses ranged from 89.2% to 111.3%, whereas Ortho Flex ranged from 8.8% to 76.8%, with the 8.0 s condition being the only Ortho Flex condition above the 70% cytotoxicity threshold. Ortho Flex showed pronounced cytotoxicity in concentrated extracts, particularly at shorter exposure times, whereas TC-85 showed a less monotonic exposure-dependent pattern. In the exploratory screening, the 6.25% Ortho Flex extract produced a normalized MCF-7 MTT response of 129.1%, but this was comparable with the corresponding saline vehicle response of 137.0% and was not statistically greater than the response in ER-negative MDA-MB-231 cells. No statistically supported ER-selective response was detected for either tested resin under the specific screening conditions; however, exposure-time dependence of this response was not evaluated. Per-layer UV exposure therefore influenced the HGF-1 MTT response in a resin- and concentration-dependent manner. For dental laboratory practice, these findings support the use of resin-specific, validated printing parameters rather than transferring exposure settings between materials; deviations from manufacturer-specified processing conditions should be independently validated before clinical use.

## 1. Introduction

Directly printed clear aligners are emerging as an alternative to conventionally thermoformed orthodontic appliances. Their additive manufacturing workflow eliminates the need for a physical model, enables direct control over appliance geometry and thickness, and may improve manufacturing efficiency and dimensional accuracy [1,2,3,4,5,6,7,8,9]. Unlike conventional thermoplastic aligners, directly printed appliances are fabricated from photocurable resins that undergo layer-by-layer photopolymerization followed by cleaning and post-curing procedures [10,11,12,13]. Consequently, their final properties depend not only on the chemical composition of the resin but also on the parameters used during printing and post-processing.

Incomplete or suboptimal polymerization may result in residual monomers, oligomers, photoinitiators, and other low-molecular-weight components remaining within the polymer network and subsequently leaching from the material [11,12,13,14,15,16]. This is particularly relevant for orthodontic aligners because they are intended for prolonged daily use in close contact with oral tissues [17]. Biocompatibility assessment of such materials therefore represents an important component of their evaluation. In vitro cytotoxicity testing is commonly performed according to the principles of ISO 10993, with ISO 10993-5:2009 providing criteria for the interpretation of cellular responses and ISO 10993-12:2021 defining procedures for sample preparation and extraction [18,19,20].

Per-layer UV exposure represents an important processing parameter because it directly affects polymerization during layer-by-layer fabrication before the final post-curing stage. In a previous study using the same Tera Harz TC-85 and NextDent Ortho Flex resins and the same printing system, per-layer UV exposure time was shown to influence the degree of conversion and thermomechanical properties of the printed materials [21]. For Ortho Flex, 7 s per layer produced the highest degree of conversion among the tested conditions, whereas both shorter (5 and 6 s) and longer (8 s) exposures resulted in significantly lower values. In contrast, TC-85 showed a comparatively stable degree of conversion across several exposure conditions, with 2.4, 3.0, and 4.5 s not differing significantly from the 4 s control [21]. The response was therefore material-specific, indicating that exposure settings cannot necessarily be generalized across different photocurable aligner resins [21]. These findings raise the question of whether processing-dependent differences in polymerization are accompanied by corresponding changes in biological response.

In addition to general cytotoxicity, potential endocrine-related effects of orthodontic polymers have received increasing attention because some polymer components or degradation products may exhibit estrogen-like biological activity [22,23,24,25]. Cell-based screening using estrogen receptor-positive MCF-7 cells may identify cellular responses compatible with estrogen-like activity, while inclusion of an estrogen receptor-negative cell line such as MDA-MB-231 can provide complementary information regarding response selectivity [22,26,27]. Such screening is exploratory and does not, by itself, demonstrate estrogen receptor activation. Increased proliferative or metabolic activity in MCF-7 cells alone cannot be considered definitive evidence of estrogen receptor-mediated activity, particularly when comparable responses are also observed in receptor-negative cells or vehicle controls.

Most available biological studies of directly printed aligner materials have evaluated materials processed according to manufacturer-recommended protocols or have focused primarily on post-printing and post-curing procedures [23,28,29,30,31,32,33,34]. Comparatively little attention has been given to the biological consequences of modifying the UV exposure applied to each individual layer during printing. Such exposure modifications represent experimental deviations from validated or manufacturer-specified processing conditions and should therefore not be interpreted as recommended clinical manufacturing protocols. Because per-layer exposure affects polymer network formation before final post-curing, variations in this parameter could also be associated with differences in the amount or composition of potentially extractable substances; however, such chemical changes were not directly investigated in the present study.

Therefore, the aim of this study was to evaluate the effect of previously characterized, resin-specific per-layer UV exposure conditions on the cytotoxicity of Tera Harz TC-85 and NextDent Ortho Flex and to conduct an exploratory screening of selected printing conditions for ER-selective proliferative/metabolic responses potentially compatible with estrogen-like activity. Human gingival fibroblasts were used for cytotoxicity assessment, whereas estrogen receptor-positive MCF-7 and estrogen receptor-negative MDA-MB-231 cells were used for exploratory ER-selective response screening. The null hypothesis was that, within each resin, per-layer UV exposure condition would not significantly affect human gingival fibroblast viability across the tested extract concentrations. The ER-selective screening component was exploratory and hypothesis-generating; because only one printing condition per resin was evaluated, it was not designed to test exposure-time dependence of potential estrogen-like responses.

## 2. Materials and Methods

### 2.1. Specimen Preparation and Extraction

Two photocurable resins intended for the direct fabrication of orthodontic appliances were investigated: Tera Harz TC-85DAC (Graphy Inc., Seoul, Republic of Korea) and NextDent Ortho Flex (Vertex-Dental B.V., Soesterberg, The Netherlands). Manufacturer-disclosed composition and relevant published analytical characterization of the resin systems are summarized in Table 1. Because commercial resin formulations are partly proprietary and published analytical studies may involve different production lots, the reported information should not be interpreted as a complete chemical characterization of the specific lots used in the present study.

Rectangular specimens were designed as cuboids with nominal dimensions of 1 mm × 3 mm × 12 mm using SolidWorks (Version 33.0.0) (Dassault Systèmes, Vélizy-Villacoublay, France). Specimens were fabricated using a SprintRay Pro95 digital light processing printer (SprintRay Inc., Los Angeles, CA, USA) equipped with a 405 nm light source. A nominal layer thickness of 100 μm was used. All specimens were positioned in a near-vertical orientation at 75° relative to the horizontal build platform, and the orientation was kept constant throughout the study.

The resin-specific per-layer UV exposure conditions were selected from those investigated in our previous physicochemical characterization using the same materials and printing system [21]. TC-85 specimens were printed at 2.0, 2.4, 3.0, and 4.0 s per layer, whereas Ortho Flex specimens were printed at 5.0, 6.0, 7.0, and 8.0 s per layer. These exposure ranges were used experimentally to determine whether previously observed exposure-dependent differences in physicochemical behavior were accompanied by differences in biological response; they should not be interpreted as manufacturer-recommended exposure settings.

For each resin × exposure condition, nine specimens were fabricated in three independent print builds, with three specimens produced for each extraction batch. Exposure conditions were printed in separate builds so that the programmed per-layer exposure time was constant within each build. The same specimen geometry, orientation, layer thickness, printer, and post-processing conditions were maintained across exposure groups.

Following printing, specimens from both materials underwent the common experimental post-processing protocol previously used with the same resin–printer system [21]. Residual uncured resin was removed by two consecutive 5 min centrifugation cycles, followed by post-curing for 20 min in a Tera Harz Cure THC2 unit (Graphy Inc., Seoul, Republic of Korea) under a nitrogen atmosphere [15,21]. Previous characterization of TC-85 identified centrifugation followed by nitrogen post-curing as the standard processing condition and demonstrated the importance of the oxygen-free curing environment [15]. For Ortho Flex, however, this common experimental post-processing protocol represented a deliberate deviation from the material-specific manufacturer workflow, which specifies alcohol-based cleaning and post-curing using the validated NextDent workflow [24]. The common protocol was used in the present experimental design to keep post-processing constant while investigating per-layer UV exposure as the principal manufacturing variable.

Following post-processing, specimens were stored in closed, light-protected sterile containers at room temperature, and extraction was initiated within 24 h. Extracts were prepared in sterile 0.9% saline according to ISO 10993-12:2021 [18]. Based on the nominal dimensions of 1 mm × 3 mm × 12 mm and inclusion of all six surfaces, each specimen had a calculated surface area of 102 mm^2^ (1.02 cm^2^). Three specimens were pooled in 1.0 mL of extraction medium for each independent extraction batch, corresponding to a surface-area-to-volume ratio of 3.06 cm^2^/mL, consistent with the ISO target ratio of approximately 3 cm^2^/mL for specimens of this thickness [18]. Three independent extraction batches were therefore prepared for each resin × exposure condition.

Specimens were completely immersed in the extraction medium and incubated at 37 °C for 24 h in the dark. Following extraction, the eluates were filtered through sterile 0.22 μm membranes before use in the cell-based assays. Sterile 0.9% saline was selected as the aqueous extraction vehicle, and concentration-matched saline vehicle controls were included in all subsequent assays to account for potential vehicle-related effects. For cytotoxicity testing, undiluted extracts and nominal extract concentrations of 50%, 25%, and 12.5% were evaluated, whereas 12.5% and 6.25% extracts were used for the exploratory ER-selective response screening.

### 2.2. Cytotoxicity Assay

Human gingival fibroblast HGF-1 cells (ATCC CRL-2014; American Type Culture Collection, Manassas, VA, USA) were used for cytotoxicity assessment. HGF-1 is an adherent human fibroblast cell line derived from normal gingival tissue. Cell-line identity was documented by the supplier using short tandem repeat (STR) profiling, and mycoplasma contamination was reported as not detected. Cells were maintained in ATCC-formulated Dulbecco’s Modified Eagle’s Medium (DMEM; ATCC 30-2002) supplemented with 10% fetal bovine serum and 1% penicillin–streptomycin (final concentrations: 100 U/mL penicillin and 100 μg/mL streptomycin) at 37 °C in a humidified atmosphere containing 5% CO_2_. Cells between passages 3 and 6 were used for all experiments.

HGF-1 cells were seeded in flat-bottom 96-well plates at approximately 8 × 10^3^ cells/well (25,000 cells/cm^2^) and allowed to attach for 24 h before treatment, consistent with the seeding density previously used for gingival-fibroblast testing of 3D-printed orthodontic aligner eluates [37].

To obtain cell-compatible test media while preserving the relative concentration of the saline extracts, each stock extract was mixed 1:1 (*v*/*v*) with double-strength DMEM containing 20% fetal bovine serum and double-strength penicillin–streptomycin. The resulting preparation contained single-strength DMEM, 10% fetal bovine serum, and standard antibiotic concentrations and was operationally designated as the 100% nominal test extract. This designation refers to the highest test concentration after cell-culture medium adjustment and not to undiluted saline eluate in the final well. The 100% nominal test extract was serially diluted with complete single-strength culture medium to obtain nominal extract concentrations of 50%, 25%, and 12.5%. Vehicle controls were prepared using sterile 0.9% saline instead of resin extract and were diluted identically, ensuring equivalent culture-medium, serum, antibiotic, and saline proportions for each corresponding extract concentration.

Following the 24 h attachment period, the culture medium was removed and replaced with 100 μL/well of the respective extract-containing test medium. Cells were exposed to the extracts for 24 h. Untreated HGF-1 cells maintained in complete culture medium served as the negative cellular control. A 0.4% Triton X-100 solution and dimethyl sulfoxide (DMSO) at final concentrations of 10%, 5%, and 2.5% were included as positive cytotoxicity controls.

Cellular metabolic activity was assessed using the MTT assay. Following 24 h of exposure, the test medium was removed, and cells were incubated with 100 μL/well of MTT working solution at a final concentration of 1 mg/mL for 4 h at 37 °C. The MTT solution was then removed, and the resulting formazan crystals were dissolved in 100 μL of 2-propanol per well. Plates were gently agitated for 10 min to ensure complete solubilization. Absorbance was measured at 595 nm using a FlexStation 3 Multi-Mode Microplate Reader (Molecular Devices, San Jose, CA, USA), following a published HGF-1 MTT protocol for 3D-printed dental materials [38]. No secondary reference wavelength was applied. After subtraction of the plate-specific mean blank absorbance, the normalized MTT response was calculated using Equation (1):

(1) $$Normalized\;MTT\;response\;\left(\%\right)=\frac{OD\;sample-OD\;blank}{OD\;untreated\;control\;-OD\;blank\;}\times 100$$

where *OD sample* represents the absorbance of the tested condition, *OD blank* the mean absorbance of cell-free reagent blanks, and *OD untreated* control the mean absorbance of untreated cells. Blank-corrected values below zero were retained without truncation as normalized MTT responses. Such values indicate absorbance below the mean blank signal and do not represent negative numbers of viable cells.

Each condition was evaluated in four technical replicate wells in each of three independent experimental runs. Technical replicates were averaged within each run before statistical analysis. Each run used a separately propagated HGF-1 culture and one independently prepared extraction batch originating from a separate print build, as described in Section 2.1. Thus, the independent experimental run, rather than the individual technical well, was considered the experimental unit (*n* = 3 per condition).

A normalized MTT response below 70% of the untreated control was interpreted as cytotoxic according to ISO 10993-5:2009 [19,20]. Because an extract-based rather than a direct-contact assay was performed, a polyethylene physical-contact reference specimen was not included.

### 2.3. Exploratory ER-Selective Response Screening

Based on the cytotoxicity findings of the present study together with the previously characterized physicochemical behavior of the same resin–printer system [21], TC-85 printed at 2.4 s per layer and Ortho Flex printed at 7.0 s per layer were selected post hoc for exploratory ER-selective response screening. The 2.4 s TC-85 condition showed the highest normalized MTT response among the tested TC-85 conditions for the undiluted extract and had previously shown physicochemical behavior comparable with the 4.0 s control condition [21]. For Ortho Flex, 7.0 s was selected because the previous physicochemical study identified this condition as producing the highest degree of conversion among the tested Ortho Flex exposure times [21], although the 8.0 s condition produced a higher fibroblast MTT response at the 50% extract concentration in the present study. The selected conditions therefore represented post hoc resin-specific conditions and were not intended to characterize exposure-time dependence of potential estrogen-like responses.

Estrogen receptor-positive MCF-7 cells (DSMZ ACC 115) and estrogen receptor-negative MDA-MB-231 cells (DSMZ ACC 732) were obtained from the Leibniz Institute DSMZ—German Collection of Microorganisms and Cell Cultures (Braunschweig, Germany). According to the supplier, both cell lines were authenticated by short tandem repeat profiling and were negative for mycoplasma contamination. MCF-7 cells are ER-positive, whereas MDA-MB-231 cells are triple-negative, including absence of ER expression. Cells between passages 3 and 8 after recovery from cryopreservation were used for the experiments. DSMZ independently confirms STR authentication, mycoplasma-negative status and ER positivity of ACC 115, while ACC 732 is confirmed as triple-negative and ER-negative.

For the screening assay, both cell lines were adapted to a common estrogen-depleted assay medium consisting of phenol-red-free DMEM supplemented with 5% charcoal-stripped fetal bovine serum and 1% penicillin–streptomycin. Cells were maintained at 37 °C in a humidified atmosphere containing 5% CO_2_ during the assay. Although the supplier-recommended routine culture conditions differ between the two cell lines, a common phenol-red-free, hormone-depleted medium was used during the experimental period to minimize exogenous estrogenic background and permit direct comparison of responses between the ER-positive and ER-negative cell models. Phenol-red-free medium with charcoal-stripped serum is a standard strategy for reducing background estrogenic stimulation in MCF-7 screening assays.

MCF-7 and MDA-MB-231 cells were seeded in flat-bottom 96-well plates at 1 × 10^4^ cells/well in 100 μL of assay medium and allowed to attach for 24 h. The medium was then replaced with fresh estrogen-depleted medium for a further 24 h adaptation period before exposure to the resin extracts. Comparable seeding densities are widely used for MCF-7/MDA-MB-231 viability and estrogen-response assays.

Extracts of TC-85 printed at 2.4 s per layer and Ortho Flex printed at 7.0 s per layer were evaluated at nominal concentrations of 12.5% and 6.25%. These concentrations were selected to reduce the influence of overt cytotoxicity on interpretation of the ER-selective screening response. Extract-containing test media were prepared by dilution of the corresponding conditioned stock extract described in Section 2.2 with complete estrogen-depleted assay medium. Concentration-matched saline vehicle controls were prepared identically so that the final proportions of culture medium, charcoal-stripped serum, antibiotics, and saline were equivalent to those in the corresponding extract-containing wells.

Cells were exposed to resin extracts for 24 h. Untreated cells maintained in estrogen-depleted assay medium served as the negative cellular control. 17β-Estradiol (E2) at 10^−9^ M and 10^−10^ M was included as a positive control for estrogen-responsive cellular activity. These concentrations are within the range known to elicit measurable responses in estrogen-sensitive MCF-7 cells under hormone-depleted conditions [22,26]. The 24 h exposure period was intentionally matched to the resin-extract exposure period; however, it is substantially shorter than the multi-day exposure typically used in conventional E-SCREEN proliferation assays [22,24]. Accordingly, the present protocol was considered an exploratory short-duration MTT-based screening assay rather than a conventional E-SCREEN assay.

Following the 24 h exposure period, cellular metabolic activity was determined using the MTT protocol described in Section 2.2. Normalized MTT responses were calculated using Equation (1) and expressed relative to the untreated control for each cell line. Four technical replicate wells were analyzed for every condition within each of three independent experimental runs, and the technical replicates were averaged before statistical analysis. Each run used separately propagated MCF-7 and MDA-MB-231 cultures and an independently prepared resin extraction batch.

The assay was interpreted as a comparative ER-selectivity screen rather than a direct measure of estrogen-receptor activation. A response preferentially increased in ER-positive MCF-7 cells relative to the corresponding ER-negative MDA-MB-231 condition, together with an appropriate MCF-7-selective response to 17β-estradiol, was considered compatible with an ER-selective estrogen-like response. Conversely, increases of similar magnitude in both cell lines, responses comparable with the corresponding saline vehicle, or isolated increases in MTT metabolic activity were not considered sufficient evidence of estrogen-receptor-mediated activity [22,24,26,27].

### 2.4. Statistical Analysis

Statistical analyses were performed using IBM SPSS Statistics version 29.0 (IBM Corp., Armonk, NY, USA). For each experimental condition, the four technical replicate wells were averaged within each independent experimental run before statistical analysis. The independent experimental run was considered the experimental unit (*n* = 3 per condition); individual technical wells were not treated as independent observations. Data are presented as mean ± standard deviation (SD), and statistical significance was set at *p* < 0.05. Because of the exploratory design and the limited number of independent experimental runs, no formal a priori power calculation was performed. Accordingly, nonsignificant comparisons were interpreted as absence of a statistically supported difference under the tested conditions and not as evidence of equivalence or absence of an effect.

Cytotoxicity data were analyzed separately for each resin because the investigated per-layer UV exposure conditions were resin-specific. Normalized MTT response was used as the dependent variable. A randomized complete-block two-way factorial analysis of variance (ANOVA) was performed with per-layer UV exposure condition and extract concentration as fixed factors, including their interaction, and experimental run as a blocking factor. Type III sums of squares were used, and partial eta squared (partial η^2^) was reported as the effect-size estimate for the main effects and interaction. When a significant interaction was detected, Bonferroni-adjusted simple comparisons were performed to compare exposure conditions within each extract concentration.

Normality of standardized model residuals was assessed using the Shapiro–Wilk test, and homogeneity of variance was evaluated using Levene’s test. When these assumptions were violated, a generalized linear model with a normal probability distribution, identity link function, and robust covariance estimator was additionally fitted as a sensitivity analysis. The primary ANOVA results were retained when the sensitivity analysis led to the same inferential conclusion.

For the exploratory ER-selective response screening, normalized MTT responses were analyzed using a blocked three-way factorial ANOVA. Cell line (MCF-7 vs. MDA-MB-231), test group (TC-85, Ortho Flex, or concentration-matched saline vehicle), and nominal extract concentration (12.5% vs. 6.25%) were included as fixed factors, together with all two-way and three-way interactions; experimental run was included as a blocking factor. Bonferroni-adjusted simple comparisons were used to compare test groups within each cell line and concentration and to compare MCF-7 with MDA-MB-231 within the corresponding experimental condition.

The 17β-estradiol positive controls were analyzed separately using a blocked 2 × 2 factorial model with cell line (MCF-7 vs. MDA-MB-231) and 17β-estradiol concentration (10^−9^ vs. 10^−10^ M) as fixed factors and experimental run as a blocking factor. Because the homogeneity-of-variance assumption was violated for these data, inference was based on a generalized linear model with a normal probability distribution, identity link function, robust covariance estimator, and Wald chi-square tests.

Blank-corrected normalized MTT responses were analyzed without censoring or truncation, including values below zero. As defined in Section 2.2, negative normalized values indicate absorbance below the mean blank signal and were not interpreted as negative cell viability.

## 3. Results

### 3.1. Cytotoxicity

The normalized MTT response of HGF-1 cells varied according to per-layer UV exposure condition and extract concentration (Table 2; Figure 1).

For TC-85, all 100% nominal extract conditions produced normalized MTT responses below the 70% cytotoxicity threshold. Mean responses were 30.6%, 61.9%, 47.2%, and 54.9% for the 2.0, 2.4, 3.0, and 4.0 s exposure conditions, respectively. Dilution of the extracts was associated with substantially higher MTT responses. At the 50% extract concentration, mean responses were 89.2%, 101.9%, 102.5%, and 111.3% for the 2.0, 2.4, 3.0, and 4.0 s conditions, respectively. At 25%, responses ranged from 99.4% to 105.3%, and at 12.5% from 78.2% to 108.5%; thus, all TC-85 exposure conditions remained above the 70% threshold at both lower extract concentrations.

For TC-85, per-layer UV exposure condition significantly affected the normalized MTT response (F(3,30) = 4.359, *p* = 0.012, partial η^2^ = 0.304), as did extract concentration (F(3,30) = 123.261, *p* < 0.001, partial η^2^ = 0.925). A significant exposure condition × extract concentration interaction was also observed (F(9,30) = 5.499, *p* < 0.001, partial η^2^ = 0.623). Bonferroni-adjusted simple comparisons showed that, for the 100% nominal extract, the response at 2.0 s was significantly lower than at 2.4 s (*p* < 0.001) and 4.0 s (*p* = 0.007). At 50%, the response at 4.0 s was significantly higher than at 2.0 s (*p* = 0.015), whereas no statistically significant differences among exposure conditions were detected at 25%. At 12.5%, the 4.0 s condition produced a significantly lower response than the 2.0, 2.4, and 3.0 s conditions (*p* = 0.001, *p* = 0.005, and *p* < 0.001, respectively).

Ortho Flex showed a more pronounced concentration-dependent reduction in normalized MTT response. For the 100% nominal extract, all four exposure conditions were below the 70% cytotoxicity threshold, with mean blank-corrected responses of −4.0%, −3.8%, −3.1%, and 6.1% at 5.0, 6.0, 7.0, and 8.0 s, respectively. The negative values reflect absorbance signals below the mean blank after blank correction and do not represent negative cell viability. At the 50% extract concentration, the 5.0, 6.0, and 7.0 s conditions remained below the 70% threshold, with responses of 16.1%, 8.8%, and 58.4%, respectively, whereas the 8.0 s condition reached 76.8%. At 25%, responses increased to 85.0%, 78.8%, 96.6%, and 105.6% for the 5.0, 6.0, 7.0, and 8.0 s conditions, respectively. All Ortho Flex exposure conditions were also above the cytotoxicity threshold at 12.5%, with mean responses ranging from 99.4% to 110.8%.

For Ortho Flex, significant effects were observed for per-layer UV exposure condition (F(3,30) = 64.881, *p* < 0.001, partial η^2^ = 0.866), extract concentration (F(3,30) = 1275.672, *p* < 0.001, partial η^2^ = 0.992), and their interaction (F(9,30) = 33.745, *p* < 0.001, partial η^2^ = 0.910). Bonferroni-adjusted simple comparisons showed no statistically significant differences among exposure conditions for the 100% nominal extracts. At 50%, responses at 7.0 and 8.0 s were significantly higher than those at 5.0 and 6.0 s (all *p* < 0.001), and the response at 8.0 s was also significantly higher than at 7.0 s (*p* < 0.001). At 25%, the 7.0 and 8.0 s conditions produced significantly higher responses than the 5.0 and 6.0 s conditions (5.0 vs. 7.0 s, *p* = 0.036; all other corresponding comparisons, *p* < 0.001), whereas no statistically significant difference was detected between 7.0 and 8.0 s (*p* = 0.178). At 12.5%, most pairwise comparisons were not statistically significant; the only significant difference was between 6.0 and 8.0 s (*p* = 0.040), with the 6.0 s condition producing the higher response.

The concentration-matched saline vehicle did not meet the ISO 10993-5 cytotoxicity criterion at any tested concentration, with mean normalized MTT responses remaining above 90%. Assumption testing showed homogeneous variances for TC-85 (Levene’s test, *p* = 0.187), although standardized residuals deviated from normality (Shapiro–Wilk *p* = 0.014). For Ortho Flex, standardized residuals did not significantly deviate from normality (Shapiro–Wilk *p* = 0.697), whereas Levene’s test indicated heterogeneity of variance (*p* = 0.001). Robust-covariance sensitivity analyses supported the same overall inferential conclusions for exposure condition, extract concentration, and their interaction for both TC-85 (Wald χ^2^(3) = 28.594, Wald χ^2^(3) = 408.311, and Wald χ^2^(9) = 75.750, respectively; all *p* < 0.001) and Ortho Flex (Wald χ^2^(3) = 302.556, Wald χ^2^(3) = 5897.428, and Wald χ^2^(9) = 866.951, respectively; all *p* < 0.001).

### 3.2. Exploratory ER-Selective Response Screening

Normalized MTT responses differed among the tested groups and extract concentrations, with the response pattern depending on cell line (Figure 2). In ER-positive MCF-7 cells, TC-85 produced mean normalized MTT responses of 2.9% and 40.2% at 12.5% and 6.25% extract concentrations, respectively, whereas Ortho Flex produced responses of 76.2% and 129.1%. The corresponding saline vehicle responses were 126.8% and 137.0%, respectively. Thus, the elevated MCF-7 response observed for Ortho Flex at 6.25% was similar in magnitude to that of the corresponding saline vehicle. In ER-negative MDA-MB-231 cells, mean responses were 32.6% and 88.4% for TC-85, 75.7% and 99.5% for Ortho Flex, and 101.0% and 105.7% for the saline vehicle at 12.5% and 6.25%, respectively.

The blocked factorial analysis showed significant effects of test group (F(2,22) = 30.114, *p* < 0.001, partial η^2^ = 0.732) and extract concentration (F(1,22) = 14.100, *p* = 0.001, partial η^2^ = 0.391), together with a significant cell line × test group interaction (F(2,22) = 6.512, *p* = 0.006, partial η^2^ = 0.372). The main effect of the cell line was not statistically significant (*p* = 0.944). Likewise, the cell line × concentration interaction (*p* = 0.667), test group × concentration interaction (*p* = 0.134), and three-way interaction (*p* = 0.608) were not statistically significant. Model assumptions were satisfied, with no statistically significant evidence of heterogeneity of variance (Levene’s test, *p* = 0.074) or deviation from normality of standardized residuals (Shapiro–Wilk W = 0.981, *p* = 0.791).

Bonferroni-adjusted simple comparisons showed that, in MCF-7 cells, TC-85 produced significantly lower normalized MTT responses than Ortho Flex and the saline vehicle at both 12.5% (*p* = 0.003 and *p* < 0.001, respectively) and 6.25% (both *p* < 0.001). Ortho Flex did not differ significantly from the corresponding saline vehicle at either 12.5% (*p* = 0.070) or 6.25% (*p* = 1.000). In MDA-MB-231 cells, TC-85 produced a significantly lower response than the saline vehicle at 12.5% (*p* = 0.010), whereas the remaining test-group comparisons at 12.5% and all comparisons at 6.25% were not statistically significant.

Direct comparisons between the two cell lines did not identify a statistically supported preferential MCF-7 response for either resin. The only statistically significant cell-line difference among the resin conditions was observed for TC-85 at 6.25%, where the normalized MTT response was lower in MCF-7 cells than in MDA-MB-231 cells (*p* = 0.030). For Ortho Flex, no statistically significant MCF-7 vs. MDA-MB-231 difference was detected at either 12.5% (*p* = 0.981) or 6.25% (*p* = 0.167).

The 17β-estradiol positive control produced a preferential response in ER-positive MCF-7 cells. Mean normalized MTT responses were 234.7% and 181.2% at 10^−9^ and 10^−10^ M 17β-estradiol, respectively, compared with 110.0% and 111.2% in MDA-MB-231 cells. Because homogeneity of variance was violated for the 17β-estradiol controls (Levene’s test, *p* = 0.007), inference was based on the generalized linear model with robust covariance estimation described in Section 2.4. Significant effects were detected for cell line (Wald χ^2^(1) = 57.335, *p* < 0.001), 17β-estradiol concentration (Wald χ^2^(1) = 4.147, *p* = 0.042), and their interaction (Wald χ^2^(1) = 4.534, *p* = 0.033). MCF-7 responses were significantly higher than MDA-MB-231 responses at both 10^−9^ M (Wald χ^2^(1) = 29.834, *p* < 0.001) and 10^−10^ M (Wald χ^2^(1) = 35.043, *p* < 0.001). Within MCF-7 cells, the response at 10^−9^ M was significantly higher than that at 10^−10^ M (*p* = 0.020), whereas no statistically significant concentration-dependent difference was detected in MDA-MB-231 cells (*p* = 0.918). Standardized residuals from the corresponding blocked model did not significantly deviate from normality (Shapiro–Wilk W = 0.925, *p* = 0.330).

Overall, no statistically supported ER-selective response to either resin extract was detected under the conditions of this exploratory screening assay. In particular, the 129.1% MCF-7 response observed for Ortho Flex at 6.25% was not significantly different from either the corresponding MDA-MB-231 response or the saline vehicle response of 137.0%, whereas the 17β-estradiol positive control produced a clear preferential response in MCF-7 cells. Given the limited number of independent experimental runs (*n* = 3), nonsignificant MCF-7 vs. MDA-MB-231 comparisons should not be interpreted as evidence of equivalence or as proof of absence of an estrogen-like effect.

## 4. Discussion

The present study showed that per-layer UV exposure time was associated with significant differences in the normalized MTT response of HGF-1 cells exposed to extracts of directly printed orthodontic aligner resins. Importantly, the magnitude and direction of this association depended on both the resin and extract concentration. Significant exposure condition × extract concentration interactions were observed for TC-85 and Ortho Flex, indicating that the biological response to changes in per-layer exposure cannot be described by a simple monotonic relationship in which longer exposure necessarily results in greater cytocompatibility. These findings complement our previous physicochemical characterization of the same resin–printer system, in which per-layer exposure influenced degree of conversion and thermomechanical behavior [21]. However, degree of conversion was not measured in the specimens used in the present biological experiments, and the relationship between physicochemical and biological outcomes should therefore be regarded as associative rather than mechanistically established.

The most pronounced exposure-dependent differences were observed for Ortho Flex. The 100% nominal extracts produced normalized MTT responses below the 70% ISO 10993-5 threshold under all four exposure conditions. At the 50% extract concentration, the 5.0 and 6.0 s conditions remained strongly cytotoxic, the 7.0 s condition produced a response of 58.4%, and only the 8.0 s condition exceeded the 70% threshold. At 25%, the 7.0 and 8.0 s conditions produced significantly higher responses than most of the shorter exposure conditions. These findings are consistent with the hypothesis that per-layer exposure may modify the amount and/or composition of extractable species; however, the extracts were not chemically characterized, and this mechanism was not directly investigated. Previous studies and reviews have similarly demonstrated that the biological response of 3D-printed dental resins can vary substantially with polymerization, washing, post-curing, and other manufacturing conditions [11,12,13,14,15,16,28,29,30,31,32,39]. A recent study of other 3D-printed dental resin composites also found acceptable HGF responses when materials were processed under optimized conditions, emphasizing that biological behavior is strongly dependent on the specific material and processing protocol rather than on additive manufacturing per se [40].

TC-85 showed a different and less pronounced exposure-dependent pattern. Although all 100% nominal TC-85 extracts were below the 70% threshold, responses increased above the threshold after dilution to 50%, 25%, and 12.5%. Exposure-related differences were present at individual concentrations but were not monotonic; for example, the 4.0 s condition produced a higher response than the 2.0 s condition at 50%, whereas at 12.5% the 4.0 s condition produced a lower response than the other TC-85 exposure conditions. These findings differ from some previous studies of TC-85, but direct comparisons are limited by substantial methodological differences. Pratsinis et al. reported no cytotoxic effect of TC-85 aligner eluates at final concentrations of 20%, 10%, and 5% following 14-day immersion in deionized water [24], whereas the present study used a different extraction vehicle, extraction ratio, exposure concentrations, specimen geometry, and manufacturing protocol. Other TC-85 studies have likewise shown that post-curing conditions can influence the HGF response [28,29]. For example, Iodice et al. observed differences in cytotoxicity among TC-85 specimens subjected to different post-curing durations, supporting the importance of processing conditions when comparing studies [29].

The resin-specific response is also biologically plausible given the differences in formulation and photopolymerization chemistry summarized in Table 1 [35,36,37]. Commercial dental photopolymers contain different oligomeric matrices, reactive diluents, photoinitiators, stabilizers, and proprietary additives, all of which may influence polymerization kinetics and the identity and quantity of extractable substances. Photoinitiator efficiency, light attenuation, oxygen inhibition, network crosslinking, and the degree of conversion of reactive carbon–carbon double bonds are known to affect polymer network formation and residual component release [41,42,43,44,45,46]. Nevertheless, the present study did not determine individual chemical constituents in the extracts, and the observed differences cannot be attributed to any specific monomer, oligomer, photoinitiator, or degradation product.

The present findings should also be interpreted in relation to our previous study of the same resins and printing system [21]. For Ortho Flex, the 7.0 s condition previously produced the highest degree of conversion among the investigated exposure conditions, whereas TC-85 showed a broader range of exposure conditions with relatively stable physicochemical behavior [21]. These previous data contributed to the post hoc selection of the 7.0 s Ortho Flex and 2.4 s TC-85 conditions for the exploratory ER-selective screening. However, degree of conversion was not measured again in the specimens used here, and the present data do not establish that differences in degree of conversion caused the observed differences in normalized MTT response. Accordingly, the results support the concept of resin-specific processing behavior but do not establish a direct physicochemical–biological causal pathway.

The exploratory ER-selective response screening yielded a more cautious finding. TC-85 did not produce a statistically supported preferential increase in MCF-7 response relative to MDA-MB-231 cells. Ortho Flex at 6.25% produced a mean normalized MCF-7 response of 129.1%; however, this value was similar to the corresponding saline vehicle response of 137.0%, and it was not significantly higher than the response in ER-negative MDA-MB-231 cells. The elevated saline-vehicle response is an important limitation of the screening assay because it demonstrates that the vehicle and/or test-medium preparation was not biologically neutral in MCF-7 cells. The mechanism underlying this increase cannot be determined from the present experiment and may reflect a transient metabolic response to the saline-containing test medium or assay variability. Consequently, resin responses should be interpreted primarily relative to the corresponding concentration-matched vehicle rather than solely relative to the untreated 100% control.

This interpretation is consistent with the principle underlying E-SCREEN-type approaches, in which an increase in MCF-7 metabolic or proliferative activity alone is insufficient to establish estrogen-receptor-mediated activity without appropriate positive, vehicle, and ER-negative comparison controls [22,24,25,26,27,33,34]. Importantly, the present 24 h MTT-based screening protocol was substantially shorter than the multi-day exposure used in conventional E-SCREEN assays and should therefore not be considered equivalent to a validated E-SCREEN. Pratsinis et al., using a longer-duration assay with TC-85, likewise reported no detectable xenoestrogenic response under their experimental conditions [24]. The agreement in direction is reassuring, but the considerable methodological differences between studies preclude direct quantitative comparison.

The behavior of the 17β-estradiol positive control supports the ability of the experimental system to detect a preferential response in ER-positive cells. At both 10^−9^ and 10^−10^ M, MCF-7 responses were significantly greater than those of MDA-MB-231 cells, whereas the resin extracts did not reproduce this pattern. Thus, no statistically supported ER-selective response was detected for either resin under the conditions of this exploratory screening assay. However, because only three independent experimental runs were performed, nonsignificant MCF-7 vs.MDA-MB-231 comparisons should not be interpreted as evidence of equivalence or as proof that estrogen-like activity is absent. Confirmation would require a larger number of independent experiments together with receptor-specific mechanistic approaches such as ER-antagonist experiments, estrogen-responsive reporter assays, or expression analysis of estrogen-responsive genes.

The present experimental design also requires distinction from validated clinical manufacturing workflows. The investigated per-layer exposure times were deliberately varied as an experimental factor, and the common post-processing protocol was used to control post-processing across exposure groups. In particular, the Ortho Flex specimens were not processed using the complete manufacturer-validated NextDent workflow [37]. Therefore, the biological responses observed here should not be interpreted as a safety assessment of Ortho Flex manufactured according to its instructions for use, nor should any individual experimental exposure condition be interpreted as a recommended clinical printing parameter. Recent biological-safety literature likewise emphasizes that 3D-printing resins should be processed according to material- and manufacturer-specific validated protocols because washing and post-curing can materially influence biological performance [39].

The static extraction model further limits translation to clinical exposure. Directly printed aligners are worn in an environment characterized by continuous salivary flow, repeated swallowing and clearance, temperature and pH fluctuations, mechanical loading, enzymatic exposure, and repeated appliance replacement [10,17]. These processes may reduce the instantaneous concentration of released substances compared with a static extract, while aging and repeated wear may generate cumulative or time-dependent release patterns not reproduced by a single 24 h extraction. Consequently, the present results characterize the response to standardized in vitro extracts and should not be interpreted as quantitative estimates of intraoral exposure.

Several additional limitations should be acknowledged. First, the extracts were not chemically characterized; therefore, the observed responses cannot be assigned to individual residual monomers, oligomers, photoinitiators, stabilizers, or degradation products. Chromatographic and mass-spectrometric analysis of the same extracts would be required to identify and quantify released compounds and relate them directly to biological responses [11,47,48]. Second, the cytotoxicity assessment relied on a single HGF-1 cell line and MTT metabolic activity, which does not capture other potentially relevant biological endpoints such as oxidative stress, inflammatory signaling, apoptosis, or genotoxicity. Third, the ER-selective component was exploratory, included only one printing condition for each resin, used a short 24 h exposure, and was based on metabolic response rather than direct measurement of receptor activation. Fourth, the saline vehicle produced elevated MCF-7 responses and therefore reduced the discriminatory capacity of the screening assay. Fifth, only three independent experimental runs were performed, limiting the precision of effect estimates and particularly the strength of conclusions based on nonsignificant comparisons. Finally, the static extraction model does not reproduce the dynamic conditions of clinical aligner use.

Within these limitations, the null hypothesis regarding the absence of an effect of per-layer UV exposure on the HGF-1 response was rejected for both resins. However, the exposure–response relationship was resin-specific and concentration-dependent rather than monotonic. The findings therefore support careful validation of resin-specific manufacturing windows rather than the assumption that increasing per-layer exposure necessarily improves biological performance. For the exploratory ER-selective screening, no statistically supported preferential MCF-7 response was detected for either selected resin condition, while the 17β-estradiol positive control produced the expected cell-line-selective response. Taken together, the findings emphasize both the importance of controlled manufacturing parameters and the need for appropriate material-specific, vehicle, and receptor-selectivity controls when evaluating the biological behavior of directly printed orthodontic resins.

## 5. Conclusions

Per-layer UV exposure time significantly influenced the normalized MTT response of HGF-1 cells exposed to extracts of both directly printed orthodontic aligner resins. The magnitude and direction of this effect depended on the resin and extract concentration and were not strictly monotonic, indicating that longer per-layer exposure does not necessarily result in a more favorable biological response. These findings support the need for resin- and printer-specific validation of manufacturing parameters rather than empirical modification of exposure settings.

In the exploratory ER-selective screening, neither the selected TC-85 nor Ortho Flex condition produced a statistically supported preferential response in ER-positive MCF-7 cells relative to ER-negative MDA-MB-231 cells. The increased MCF-7 response observed for Ortho Flex at 6.25% was comparable with the corresponding saline vehicle response, whereas 17β-estradiol produced the expected preferential MCF-7 response. Thus, no statistically supported ER-selective response was detected under the tested conditions. Given the short-duration exploratory design and the limited number of independent experimental runs, these findings should not be interpreted as proof of absence of estrogen-like activity.

Overall, the results emphasize that the biological response of directly printed orthodontic resins depends on both material composition and manufacturing conditions and that deviations from validated resin-specific workflows require independent biological evaluation before their clinical relevance can be established.

## Figures and Tables

**Figure 1:**
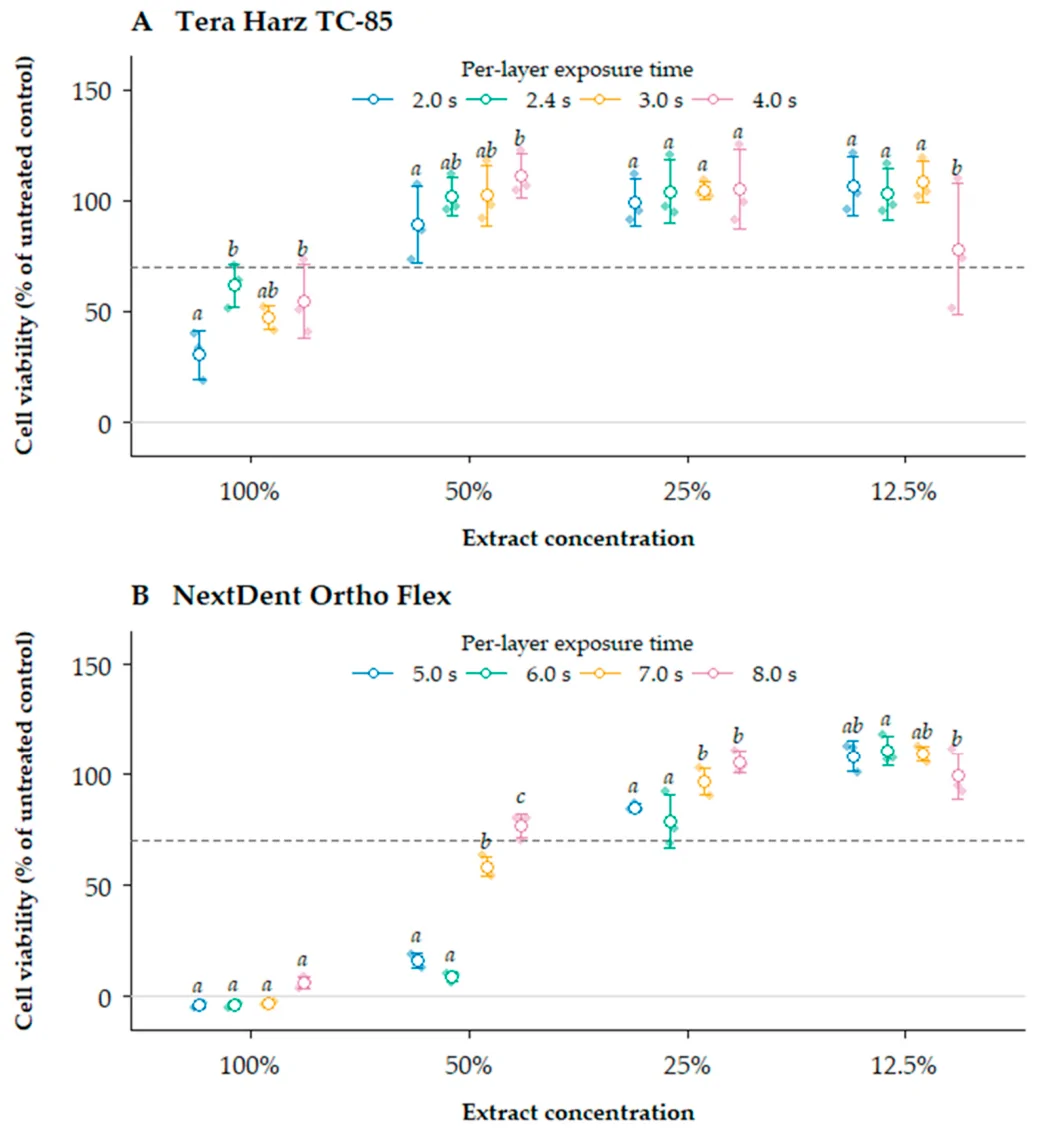
Normalized MTT response of HGF-1 human gingival fibroblasts after 24 h exposure to extracts of directly printed aligner materials fabricated using different per-layer UV exposure conditions: (**A**) Tera Harz TC-85 and (**B**) NextDent Ortho Flex. Responses are expressed relative to untreated control cells. Large open symbols and error bars represent mean ± SD from three independent experimental runs, whereas smaller symbols represent the individual experimental runs. Different lowercase letters indicate statistically significant differences among per-layer exposure conditions within the same extract concentration based on Bonferroni-adjusted simple comparisons (*p* < 0.05). The dashed horizontal line indicates the 70% cytotoxicity threshold according to ISO 10993-5:2009.

**Figure 2:**
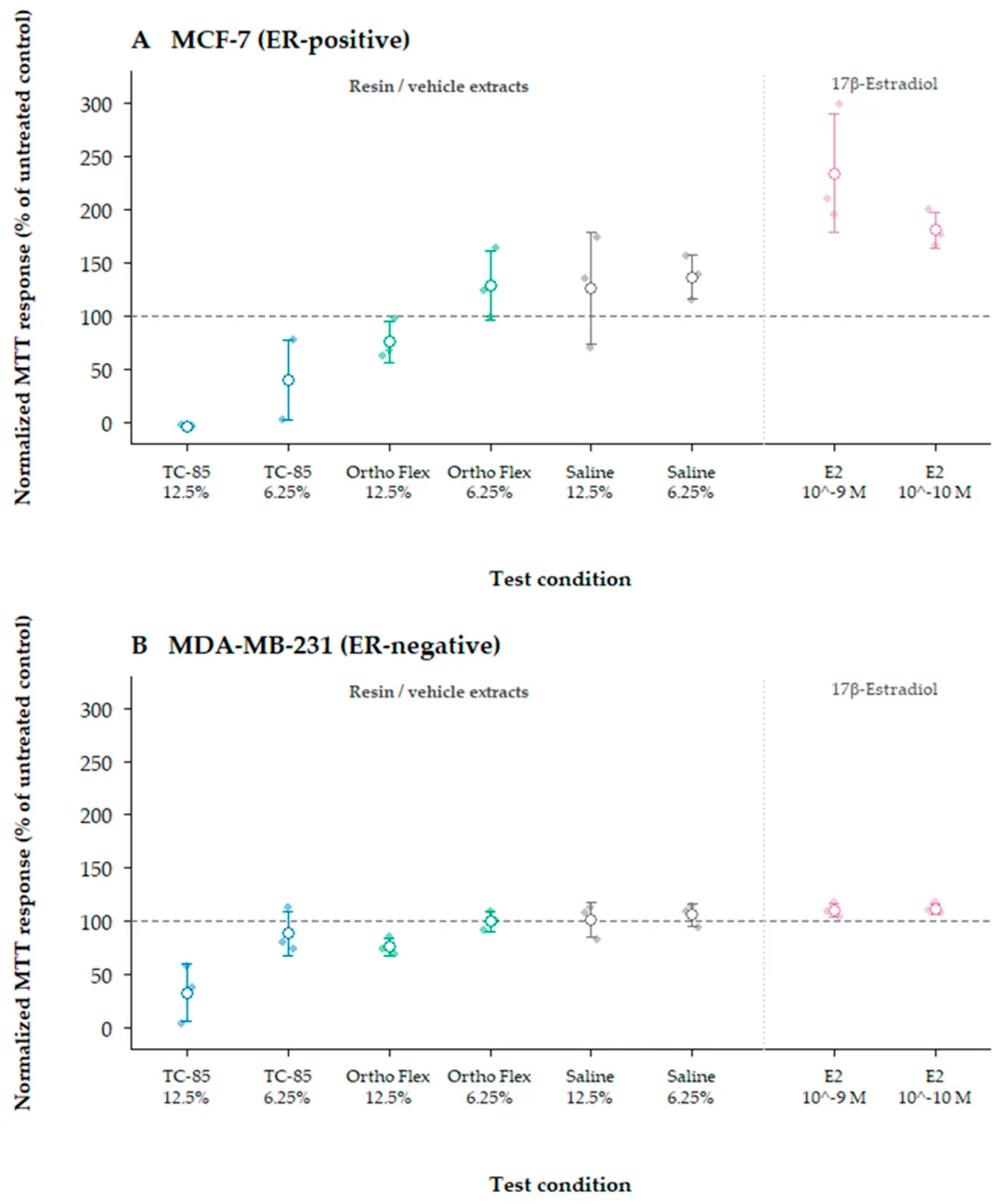
Normalized MTT responses of ER-positive MCF-7 and ER-negative MDA-MB-231 cells after 24 h exposure to extracts of Tera Harz TC-85 printed at 2.4 s per layer, NextDent Ortho Flex printed at 7.0 s per layer, concentration-matched saline vehicle controls, and 17β-estradiol positive controls. (**A**) MCF-7 cells; (**B**) MDA-MB-231 cells. Large open symbols and error bars represent mean ± SD from three independent experimental runs, whereas smaller symbols represent the individual experimental runs. The dashed horizontal line indicates the untreated-control response (100%).

**Table 1:** Investigated photocurable resins, manufacturer-disclosed composition, and published analytical characterization.

Material	Manufacturer	LOT	Manufacturer-Disclosed Composition	Published Analytical Characterization
Tera Harz TC-85DAC	Graphy Inc., Seoul, Republic of Korea	1-DL15B01066	The SDS lists four proprietary formulation ingredients and a photoinitiator; individual chemical identities are not disclosed [35].	Previous FTIR characterization demonstrated polyurethane- and acrylate-associated functional groups [15]. Lee et al. reported GR30860 and GR3060 oligomer resins and bis(2,4,6-trimethylbenzoyl)-phenylphosphine oxide (BAPO; Irgacure 819) as the photoinitiator [36].
NextDent Ortho Flex	Vertex-Dental B.V., Soesterberg, The Netherlands	WW115N01	2-Phenoxyethyl acrylate; 4-(1-oxo-2-propenyl)-morpholine; methacrylate ester monomer; diphenyl(2,4,6-trimethylbenzoyl)phosphine oxide (TPO); acrylate ester [37].	FTIR characterization of Ortho Flex identified urethane-associated N–H and amide C=O signals, ester C=O, aliphatic C=C, C–O–C/C–C–O bands, and aromatic functional-group signals [21].

**Table 2:** Normalized MTT response (%) of HGF-1 human gingival fibroblasts after 24 h exposure to extracts of directly printed aligner materials fabricated using different per-layer UV exposure conditions.

Material	Exposure (s)	100% Nominal Extract	50% Extract	25% Extract	12.5% Extract
Tera Harz TC-85	2.0	30.6 ± 11.1	89.2 ± 17.2	99.4 ± 10.8	106.7 ± 13.1
	2.4	61.9 ± 9.7	101.9 ± 8.9	104.2 ± 14.4	103.2 ± 11.7
	3.0	47.2 ± 5.5	102.5 ± 13.6	104.8 ± 4.0	108.5 ± 9.4
	4.0	54.9 ± 16.8	111.3 ± 9.9	105.3 ± 17.8	78.2 ± 29.5
NextDent Ortho Flex	5.0	−4.0 ± 1.1	16.1 ± 3.1	85.0 ± 1.7	108.4 ± 6.7
	6.0	−3.8 ± 1.3	8.8 ± 2.2	78.8 ± 12.3	110.8 ± 6.6
	7.0	−3.1 ± 0.6	58.4 ± 4.6	96.6 ± 6.2	109.3 ± 3.4
	8.0	6.1 ± 3.0	76.8 ± 5.6	105.6 ± 4.9	99.4 ± 10.3
Saline vehicle	—	96.0 ± 16.8	113.0 ± 10.0	106.8 ± 6.7	112.4 ± 9.4

Values are mean ± SD from three independent experimental runs; four technical replicate wells were averaged within each run (*n* = 3 per condition). A normalized MTT response below 70% of the untreated control was interpreted as cytotoxic according to ISO 10993-5:2009. Blank-corrected values below zero were retained without censoring; these values indicate absorbance below the mean blank signal and do not represent negative cell viability.

## Data Availability

The data presented in this study are available on request from the corresponding author due to privacy and ethical restrictions of the School of Dental Medicine, University of Zagreb.
